# Relationships Between Subjective and Objective Measures of Listening Accuracy and Effort in an Online Speech-in-Noise Study

**DOI:** 10.1097/AUD.0000000000001662

**Published:** 2025-03-21

**Authors:** Ian M. Wiggins, Jemaine E. Stacey, Graham Naylor, Gabrielle H. Saunders

**Affiliations:** 1 National Institute for Health and Care Research (NIHR) Nottingham Biomedical Research Centre, Hearing Theme, Nottingham, United Kingdom; 2 Hearing Sciences, Mental Health and Clinical Neurosciences, School of Medicine, University of Nottingham, Nottingham, United Kingdom; 3 University of Leicester, Psychology & Vision Sciences, Leicester, United Kingdom; 4 Hearing Sciences (Scottish Section), Mental Health and Clinical Neurosciences, School of Medicine, University of Nottingham, Glasgow, United Kingdom; 5 Manchester Centre for Audiology and Deafness, University of Manchester, Manchester, United Kingdom.

**Keywords:** Hearing loss, Listening effort, Speech perception

## Abstract

**Objectives:**

Speech-in-noise performance is of paramount importance to daily function, and there exists a bewildering array of outcome measures to capture the many dimensions of this concept. The aim of the present study was to provide insight into how different speech-in-noise outcome measures relate to one another, how they behave under different test conditions, and how researchers or practitioners might go about selecting an outcome measure (or measures) depending on the context and focus of their enquiry.

**Design:**

An online speech-in-noise study was conducted using the Labvanced experimental platform. A total of 67 participants (42 who reported having normal hearing, 25 who said they had some degree of hearing loss) completed the Effort Assessment Scale (a self-reported measure of daily-life listening effort), followed by a sentence recognition task in which BKB sentences were presented in speech-shaped noise at signal to noise ratios (SNRs) of −8, −4, 0, +4, +8, and +20 dB. Participants were instructed to listen to each sentence and then repeat aloud what they heard. Responses were recorded through participants’ webcams and later independently scored by 2 research assistants. Several outcome measures were used to tap into both accuracy and listening effort. Specifically, we examined: (1) objective intelligibility (percentage of keywords correctly repeated); (2) subjective intelligibility; (3) subjective listening effort; (4) subjective tendency to give up listening; and (5) verbal response time (VRT) extracted from the audio recordings. Data were analyzed using Bayesian statistical methods.

**Results:**

Hearing loss and age were associated with speech-in-noise outcomes. Specifically, we observed lower intelligibility (objective and subjective), higher subjective listening effort, and longer VRT (time to verbal response onset) in hearing-impaired compared with normal-hearing listeners, and reduced objective intelligibility and longer VRT in older compared with younger listeners. When moving from highly favorable to more adverse listening conditions, subjective listening effort was the first measure to show sensitivity to worsening SNR, followed by subjective intelligibility, objective intelligibility, subjective tendency to give up listening, and, finally, VRT. Participants, especially those with normal hearing, consistently underestimated their own performance.

**Conclusions:**

The present findings offer useful insight into how different subjective and objective measures of listening accuracy and effort respond to variation in hearing status, age, and SNR. Although speech intelligibility remains a measure of primary importance, it is a sensitive measure only under adverse listening conditions, which may not be representative of everyday listening. Under more ecologically relevant listening conditions (generally speaking, at moderate, positive SNRs), listening effort becomes a crucial factor to consider to adequately describe the listening experience. VRT may provide a useful objective marker of listening effort, but caution is required to deal with measurement variability, differences in definition, and the potentially confounding effect of age.

## INTRODUCTION

Difficulty understanding speech in noise has long been recognized as a primary challenge faced by listeners with hearing loss (HL), and the topic has therefore been a mainstay of audiological research and practice (Billings et al. 2024). Historically, the focus tended to be on speech intelligibility: what proportion of the speech is a listener able to recognize correctly in a given level of noise? Or, in the context of an adaptive speech reception threshold paradigm, how much noise is a listener able to tolerate while still recognizing a certain proportion of the speech correctly? Speech intelligibility has also been queried subjectively using a variety of standardized and nonstandardized self-report measures (Akeroyd et al. 2014; Granberg et al. 2014) and, in limited cases, objective and subjective measures of speech intelligibility have been collected under identical conditions and compared (Saunders & Forsline 2006).

More recently, there has been an explosion of interest in the concept of listening effort because of the growing appreciation that correctly understanding speech is not the sole relevant factor: it is also important to consider the cognitive cost of doing so (McGarrigle et al. 2014; Pichora-Fuller et al. 2016). This has led to exploration of a plethora of subjective, behavioral, and physiological approaches to measuring listening effort. Inconveniently for hearing researchers and practitioners, the many putative measures of listening effort have generally been found to be poorly, and, at times, inconsistently, correlated with one another (Zekveld et al. 2010; Strand et al. 2018; Alhanbali et al. 2019; Lau et al. 2019; Visentin et al. 2022; Shields et al. 2023). The emerging consensus is that this is because listening effort is a complex, multi-dimensional construct, with different measures tapping into different dimensions (Alhanbali et al. 2019; Francis & Love 2020; Shields et al. 2023).

Subjective measures of listening effort generally involve participants rating how much effort they felt they had to expend to complete a listening task. Some hearing researchers have borrowed response scales from other domain-general measures of workload and exertion, such as the NASA Task Load Index (Hart & Staveland 1988) or the Borg CR-10 scale (Borg 1990), while others have used bespoke questionnaire items (Zekveld et al. 2010) or scaling methods (Krueger et al. 2017). Patient-reported outcome measures have also been developed to capture the amount of listening effort that people experience in day-to-day life (Alhanbali et al. 2017; Hughes et al. 2017, 2019).

Behavioral measures of listening effort take a variety of forms. Dual-task measures involve measuring performance on a secondary task that is administered in parallel with a primary listening task. Following the lead of Kahneman’s (1973) Capacity Model for Attention, the rationale for these measures is that a person’s overall capacity to exert cognitive effort is limited, and so if the primary listening task requires greater effort, then there will be less effort to spare for the secondary task, and consequently performance on the secondary task will suffer. The nature and sensory modality of the secondary task has varied across studies (Gagné et al. 2017; Strand et al. 2018). Recall measures operate on the assumption that memory for what has been heard will be poorer if greater effort was needed during the listening phase (Rabbitt 1968; McCoy et al. 2005). Several variants of the recall paradigm have been developed specifically for measuring listening effort (Sarampalis et al. 2009; Ng et al. 2013; Keidser et al. 2015; Lunner et al. 2016). Verbal response time (VRT) measures operationalize listening effort as an increase in the time taken to prepare and execute a verbal response in a listening task, on the basis that increased cognitive processing needed to recover meaning from a degraded speech signal will take a finite amount of time (Gatehouse & Gordon 1990; Houben et al. 2013; Pals et al. 2015). Several studies have been published in recent years in which VRT was used to measure changes in listening effort associated with a diverse range of experimental manipulations, including varying signal to noise ratio (SNR), digital noise reduction, amplification, masker type, presence of auditory distractors, age, hearing status, and language background (Gustafson et al. 2014; Meister et al. 2018; McGarrigle et al. 2019; Oosthuizen et al. 2021; Ibelings et al. 2022; Visentin et al. 2022; Gustafson et al. 2023a, b).

Physiological measures of listening effort aim to provide direct insight into the cognitive mechanisms involved in, and/or biological consequences of, exerting mental effort to achieve a listening goal. This is achieved by quantifying brain activity associated with effortful listening using a brain-imaging technique such as functional magnetic resonance imaging (Wild et al. 2012), functional near-infrared spectroscopy (Wijayasiri et al. 2017; Shatzer & Russo 2023), or electroencephalography (Wisniewski et al. 2015; Bernarding et al. 2017; Miles et al. 2017), or measuring some other biological signal as a marker for listening-related arousal/stress. By far the most common approach has been to use pupillometry, with increased pupil dilation taken as a marker of listening effort (Kramer et al. 1997; Zekveld et al. 2010; Wang et al. 2018; Winn & Moore 2018). Other researchers have examined alternative physiological signals as markers of listening effort, for example, heart-rate variability and/or skin conductance (Mackersie et al. 2015; Seeman & Sims 2015; Slade et al. 2021). As noted by Richter et al. (2023), the relationships between these various physiological parameters are complex, making it challenging to interpret data from each alone or in combination.

With choices to be made between a focus on performance (i.e., objective speech intelligibility), subjective intelligibility, or the different dimensions of listening effort, it is challenging to know which outcome measure(s) to use in a research study or in the clinic. In the present study, our aim was to provide a dataset that could help guide such decisions. Specifically, our aim was to measure outcome measures that tap into both performance and listening effort, in the same individuals and at the same moment in time, and at task difficulties that span the range of SNRs typically encountered in daily life (Smeds et al. 2015; Wu et al. 2018). The relationships between these measures, and their sensitivity to effects of hearing status, age, and SNR, provide insights that could help guide outcome-measure selection in the future.

The present study was conducted during the coronavirus disease 2019 pandemic, at a time when in-person testing was not permitted. We therefore focused on outcome measures that could be collected within the context of an online study. We recorded participants’ verbal responses via webcam and assessed the following outcome measures: objective (percentage of sentence keywords correctly recognized) and subjective speech intelligibility, subjective measures of listening effort and tendency to give up listening, and VRT as an objective, behavioral measure of listening effort. Besides the online implementation, the novelty of the study lies in the use of multivariate Bayesian statistical methods to combine measures of speech intelligibility and listening effort, in pursuit of a more holistic understanding of the difficulties individuals face when listening to speech in background noise.

## MATERIALS AND METHODS

### Participants

Participants were recruited from the Nottingham Biomedical Research Centre Hearing Theme’s volunteer database. A total of 114 visits were made to the study webpage during the recruitment period (October to December 2020), with 67 participants completing the experiment in full. Of those who completed the experiment, 36 were male, 31 were female, and the age range was 20 to 84 years old (M = 44.0, SD = 19.5). The study received ethical approval from the University of Nottingham Faculty of Medicine and Health Sciences research ethics committee, and participants were offered a £10 online retail voucher for completing the study.

Based on responses given to a self-reported hearing status questionnaire (described in Questionnaires), we divided the participants into 2 groups: people with normal hearing (NH) (N = 42, 19M/23F, median age 30, range 23 to 71 years old), who reported having no known problems with their hearing in either ear, and people with HL (N = 25, 17M/8F, median age 61, range 20 to 84 years old), who reported varying degrees of HL in one or both ears. Of those with HL, 11 were users of bilateral hearing aids, and 2 were users of a unilateral cochlear implant.

### Auditory Stimuli

During the online speech-in-noise test, participants heard BKB sentences (Bench et al. 1979) spoken by a male talker presented in steady speech-shaped noise at 6 different SNRs (−8, −4, 0, 4, 8, and 20 dB). The BKB corpus comprises short, simple sentences (e.g., “The clown had a funny face”); these are the original sentences that were adapted for American-English use in the Hearing in Noise Test (Nilsson et al. 1994). The spectrum of the speech-shaped noise was matched to the specific sentence corpus in use, to ensure consistent and effective masking. Stimuli were prepared in advance using a custom MATLAB script and uploaded to the online experimental platform as “.wav” files. Because all participants were presented with the same pre-mixed speech-in-noise recordings, consistency of the SNR (though not the absolute sound pressure level) was ensured across participants. The sampling rate was 44.1 kHz and the noise started 0.5 seconds before sentence onset and finished 0.3 seconds after sentence offset (noise onset/offset ramp duration was 200 msec). An auditory cue (50-msec 1 kHz tone burst) was appended to the end of each stimulus to alert participants when a verbal response was required.

### Questionnaires

Before the speech-in-noise test, participants completed a basic demographics and hearing status questionnaire (Supplemental Digital Content 1, http://links.lww.com/EANDH/B644), followed by the Effort Assessment Scale (EAS) (Alhanbali et al. 2017). The EAS comprises six items enquiring about perceived listening effort in different everyday communication scenarios, with ratings given on a 0 to 10 visual analog scale. Similar 0 to 10 visual analog scales were additionally used to collect subjective ratings of listening experiences at intervals throughout the speech-in-noise test. Specifically, at the end of each block of sentences, participants were asked to respond to the following three questions: (1) How much effort was needed to understand the sentences? (Anchors “No effort” and “Extreme effort”); (2) How many of the sentences did you understand? (Anchors “None” and “All”); and (3) How often did you give up trying to understand the sentences? (Anchors “Never gave up” and “Always gave up”). No numerical labels were shown on the response scales for these questions, to avoid possible confusion over whether the numbers were to be interpreted as relative proportions or as a direct count of sentences.

### Procedure

The experiment was implemented using a commercial online platform (www.labvanced.com). After completing the preliminary questionnaires, participants were given some technical instructions and completed set-up tasks to ensure their computer speakers, microphone and webcam were functioning correctly. First, participants were asked to tick a box to confirm they were completing the experiment in a quiet room where they were unlikely to be disturbed. Participants who used hearing devices were instructed to wear them as usual during the experiment, and all participants were instructed to perform the experiment while listening through computer/tablet loudspeakers (not through headphones). We encouraged free-field presentation in this way primarily because it would allow the original stimulus playback (including the tone burst that was used as a timing reference) to be captured in the webcam recordings. A sound check was conducted in which participants were presented with a sample sentence and asked to adjust the volume of their computer/tablet loudspeakers to a comfortable level. Finally, a webcam check was performed, which involved making a recording of the participant repeating back a sentence, which the participant then reviewed themselves to confirm that they could both see and hear themselves clearly in the recording.

During the speech-in-noise test, participants were required to listen to sentences in noise and respond by repeating out loud what they had heard. Each trial began with a white screen with a black “get ready” prompt displayed centrally for 1500 msec. The prompt then disappeared and the auditory stimulus was played. Timed to coincide with the auditory cue (tone burst) at the end of the stimulus, a “Speak now” prompt appeared along with a button labeled “When you are finished speaking click here.” Clicking the button moved the system to the next trial. A blank screen was displayed for 1000 msec between trials.

The speech-in-noise test comprised 4 practice trials (results discarded), followed by 6 blocks of 16 sentences each (6 × 16 = 96 trials total). The sentences within each block were presented at a fixed SNR, with block presentation order randomized across participants. After each block, the subjective ratings questionnaire was presented, followed by a pause screen which reminded participants of the key instructions and provided the option of a break before entering the next block. The entire experiment took approximately 35 minutes to complete.

### Data Analysis

A custom scoring app with a graphical user interface was developed in MATLAB to facilitate data analysis (see Figure, Supplemental Digital Content 2, http://links.lww.com/EANDH/B645). The app processed the webcam audio recordings to obtain an initial estimate of the timing of the auditory cue and the onset and offset of the participant’s verbal response. The timing of the auditory cue was estimated by performing a cross-correlation of the trial recording with the original auditory cue stimulus and identifying the location of the peak in the cross-correlation function. The timing of the response onset and offset were estimated using a robust voice activity detection algorithm (Tan et al. 2020) to detect the presence of speech in the section of the recording after the auditory cue.

Two research assistants used the scoring app to independently review all 6432 trials, in each case: (1) checking and, if necessary, adjusting the position of the audio markers*; (2) scoring the sentence keywords as correct or incorrect†; and (3) adding relevant notes such as “No attempt at keywords.” Trials on which the two scorers disagreed on keyword scoring were reviewed by author I.M.W to resolve discrepancies. Similarly, trials on which the position of the audio markers differed between scorers by more than 100 msec for time-to-auditory-cue or response-onset time, and by 200 msec for response-offset time were reviewed by author I.M.W. to resolve discrepancies; otherwise, the positions of the audio markers were simply averaged across the 2 scorers.

Statistical analyses were performed in R (R Core Team 2016) using the “BRMS” package for Bayesian statistics (v2.18.0; Bürkner 2017). EAS scores were compared between the NH and HL groups using the Gaussian distribution (Bayesian equivalent of an independent samples *t* test). In modeling the outcomes of the speech-in-noise test, our guiding approach was to stay as “close to the data as possible,” by modeling at the lowest level available. That meant modeling objective intelligibility (count of keywords correctly identified) and VRT data (time-to-response-onset and response duration) at the individual-trial level, while subjective ratings data were modeled at the block level (because these data were only captured at the end of each block of sentences). We used the beta-binomial distribution to model counts of correctly identified keywords and the log-normal distribution to model trial-level VRTs, because the latter take strictly positive values. In our primary analyses, we included VRTs only from trials on which all keywords were repeated correctly. This was done to ensure a level of consistency in the nature of the verbal responses being assessed; the implications of this decision are addressed in the Discussion. To account for differences in stimulus duration across trials, all response-time data were expressed as a multiple of the original stimulus duration.

For the subjective-ratings data, we used the ordered beta regression model by Kubinec (2023), a parsimonious model for continuous variables having lower and upper bounds ([0–10] in the case of our subjective-rating scales). This model uses a cut-point technique to simultaneously model degenerate responses at the extreme ends of the scale alongside continuous responses at intermediate locations. We found in preliminary testing on our dataset that it was much easier to achieve successful model convergence using the ordered beta regression approach than it was using zero-and-one-inflated beta regression, a popular alternative approach to modeling data from visual analog scales (Liu & Kong 2015).

For all models, we used broad, uninformative priors and ran 4 independent Markov chain Monte Carlo (MCMC) chains with 8000 iterations each (2000 warm-up iterations, 6000 sampling iterations). Successful model convergence was verified by confirming that all R-hat diagnostic values were ≤1.01 (R-hat = 1 at full convergence).

Our primary analysis tested for effects of self-reported hearing status, that is, “group” (NH versus HL) and SNR on each outcome measure, while controlling for age. Age was included as a covariate because the average age of the participants with HL was approximately twice that of those with NH, and so if not controlled for age would have been a potential confound. Accordingly, group, SNR, the Group × SNR interaction, and age were included as fixed effects in model formulae. We constructed a multivariate model such that all outcome measures could be evaluated within the same set of MCMC chains. The use of a multivariate approach also allowed us to explicitly model the correlation structure between outcome measures. This was achieved by including correlated random effects of participant, which we allowed to vary across SNR, and with different hyperparameters (SDs) of the random effects allowed within each group (to reflect anticipated differences in within-group homogeneity between the NH and HL groups). BRMS model formulae are provided for reference (Supplemental Digital Content 3, http://links.lww.com/EANDH/B646). Effects of group and SNR were assessed by calculating conditional effects (median and 95% credible interval) for the expected value of the outcome measure for each combination of group and SNR, adjusted to a grand average mean age of 44 years.

To examine the interrelationships between outcome measures, we first assessed the random-effect correlations arising from the multivariate model. Because the correlation structure was modeled explicitly, we had access to the full posterior distributions for the correlation parameters and so were able to quantify uncertainty in the estimated correlations. To provide further insight into the relationship between objective and subjective intelligibility, we further examined the correlation between the raw data for these measures using Pearson correlations and scatter plots. Finally, to gain an overall picture of how the different outcome measures varied with SNR, we treated SNR as a continuous variable and fitted third-order orthogonal polynomial models across SNR. This analysis was conducted on pooled data across the two groups, because the focus here was on general relationships between outcome measures, not on any between-group differences. As for the primary analysis, all outcome measures were evaluated within the same set of MCMC chains using a multivariate model formulation.

## RESULTS

Data from all 67 participants were included in the analyses. However, due to problems with some of the webcam recordings that precluded accurate identification of VRTs, VRT data were available for only 51 participants (34 NH, 17 HL). VRT data for the remaining 16 participants (8 NH, 8 HL) were treated as missing.

### Self-Reported Listening Effort in Daily Life

**Fig. 1.**
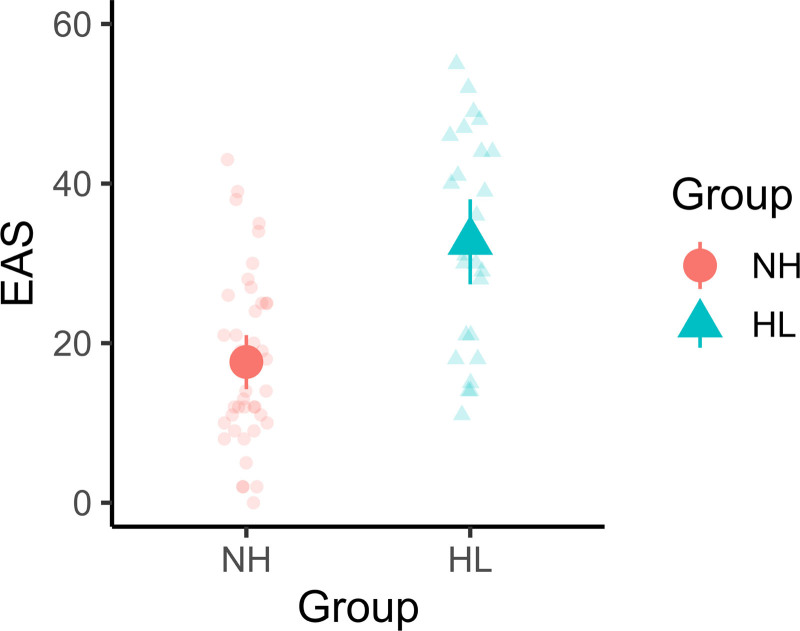
EAS scores by group—NH (circles) vs. HL (triangles). Bold symbols with error bars show medians and 95% credible intervals of the expected posterior values from the Bayesian analysis. Individual-level raw data are shown as lightly shaded symbols. EAS indicates Effort Assessment Scale; HL, hearing loss; NH, normal hearing.

Figure 1 shows EAS scores separated by self-reported hearing status (NH and HL). The average EAS score was higher for people with HL compared with those with NH (median scores 17.6 and 32.9 for the NH and HL groups, respectively; credibility of difference >99.9%). Nevertheless, inter-individual variability in EAS scores was pronounced in both groups, with substantial overlap in the range of scores observed between groups.

### Effects of Hearing Status and SNR on Objective and Subjective Outcome Measures

**Fig. 2.**
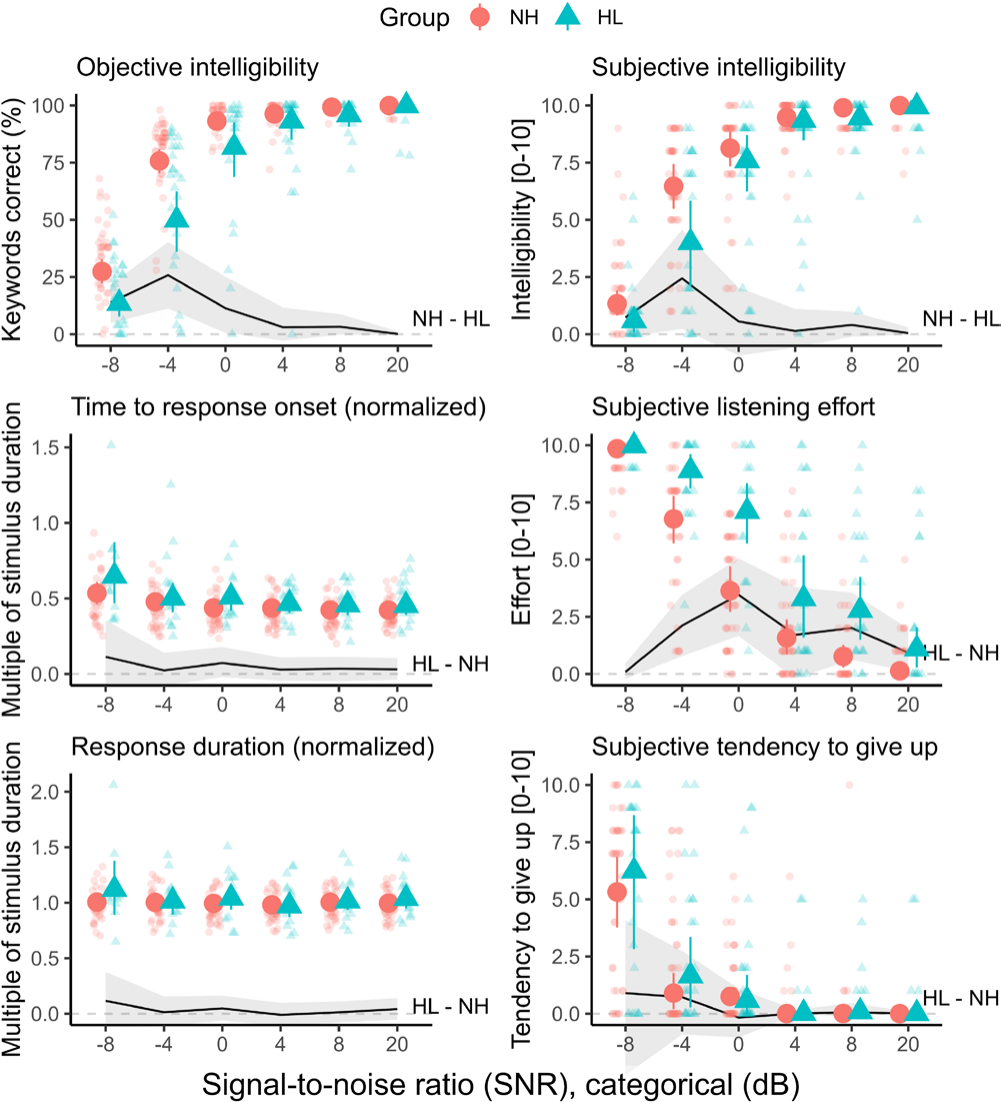
Results of primary analysis testing for effects of group—NH (circles) vs. HL (triangles)—and SNR on each outcome measure (controlling for age). Plots in the left column correspond to objective measures, while those in the right column correspond to subjective measures. Bold symbols with error bars show medians and 95% credible intervals of the expected posterior values. Individual-level raw data are shown as lightly shaded symbols. The black line with shaded error region toward the bottom of each plot shows the between-group difference, NH–HL or HL–NH as indicated (median plus 95% credible interval). HL indicates hearing loss; NH, normal hearing; SNR, signal to noise ratio.

Figure 2 shows the results of the primary analysis testing for effects of group (NH versus HL) and SNR on each outcome measure, while controlling for age. We describe the results for each outcome measure in the following paragraphs.

#### Objective Intelligibility

For both groups (NH and HL), average performance was close to or at ceiling level (100% correct) at SNRs of +4 dB and above, although 2 participants who reported severe HL had lower performance (~75% correct) even at the most favorable SNRs. Performance declined steeply as the SNR became more adverse (<0 dB), with the HL group being affected more severely than the NH group at 0 dB SNR and below (credibility of between-group difference ≥99% at 0, −4, and −8 dB SNR).

#### Subjective Intelligibility

On average, participants reported they could understand all (or almost all) sentences (scores near 10/10) at +20 dB SNR, while by −8 dB SNR, most participants reported not being able to understand any of the sentences (scores near 0/10). Interindividual variability was substantial in both the NH and HL groups. Although ratings from participants with HL were generally lower than those from participants with NH, a clear between-group difference (credibility of effect ≥95%) was evident only at the most adverse SNRs of −4 and −8 dB.

#### Subjective Listening Effort

Most participants reported that listening required no (or almost no) effort at +20 dB SNR (scores near 0/10), but extreme effort at −8 dB SNR (scores near 10/10). A minority (20%) of participants with HL rated subjective listening effort at ≥5/10 even at the most favorable SNR of +20 dB. Despite widespread variability between individuals, ratings from participants with HL were on average higher (indicating a need for more effort) than those of the NH group, with a clear between-group difference (credibility of effect ≥95%) being evident at all SNRs apart from −8 dB.

#### Subjective Tendency to Give Up

Only a small proportion of individuals reported ever giving up listening (scores >0/10) at positive SNRs (~6% of individuals at +20 dB SNR rising to ~13 to 15% at +8 and +4 dB SNR). As the SNR became negative, more individuals reported giving up, more often. At −8 dB SNR, participants reported giving up around half of the time on average (mean score = 5.5/10), although around 10% of participants reported always giving up (score of 10/10) under such challenging listening conditions. There were no clear differences between the NH and HL groups.

#### Time to Verbal Response Onset

Time to verbal response onset (for correct trials) was relatively stable across SNRs, rising by 26% (NH group; from 0.42 to 0.53 times the original stimulus duration) to 44% (HL group; from 0.45 to 0.65 times the original stimulus duration) when moving from the most favorable SNR (+20 dB) to the most adverse (−8 dB). Listeners with HL were on average slightly slower to begin their verbal response than NH listeners, but there was no clear between-group difference (credibility of effect ≥95%) at any individual SNR.

#### Duration of Verbal Response

Verbal response duration (the average amount of time for which participants were actively speaking on correct trials) remained stable at around one times the original stimulus duration (i.e., participants tended to repeat back the sentences at the same speech rate as the original stimulus recordings). There were no clear effects of SNR, nor differences between the NH and HL groups.

### Effects of Age on Objective and Subjective Outcome Measures

**Fig. 3.**
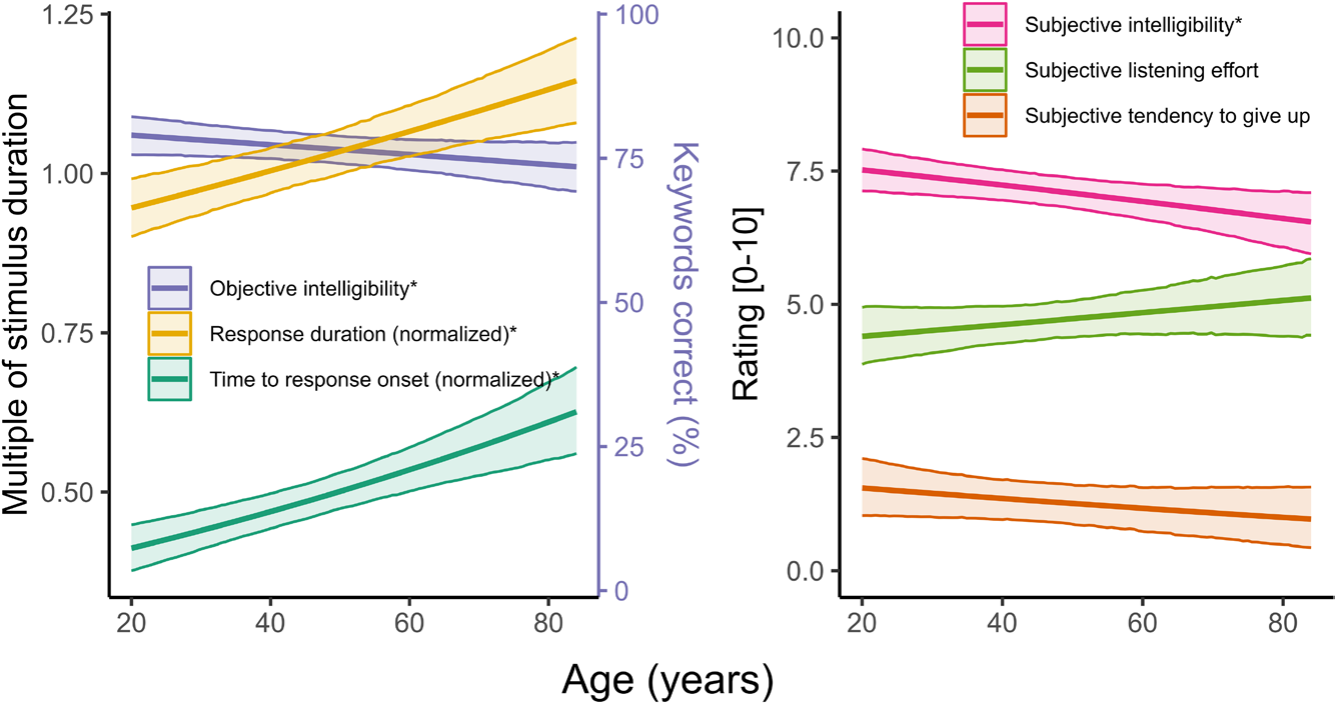
Effects of age on objective (left panel) and subjective (right panel) outcome measures, estimated from the primary analysis models. The plotted data show the median and 95% credible intervals for the expected value of each outcome measure as a function of age, averaged across groups and SNR. *≥95% credibility of an age effect (i.e., a difference in that outcome measure between a person aged 20 and 84 yr old, all else being held equal). SNR indicates signal to noise ratio.

Figure 3 shows the model-predicted overall effects of age for each outcome measure, averaged across groups (NH and HL) and SNR. A clear effect of age (≥95% credibility of difference between hypothetical participants aged 20 and 84 years old, all else being held equal) was found for all objective outcome measures: central estimates showed a decline in objective intelligibility from 79% correct to 74% correct between the ages of 20 and 84 years old; between the same ages, time to verbal response onset and verbal response duration increased by 52% and 21%, respectively, indicating that, with age, participants both became slower to start speaking and spoke for longer. Regarding the subjective outcome measures, the trend was toward increased listening effort, decreased intelligibility, and decreased tendency to give up with greater age; only for subjective intelligibility was the credibility of the effect ≥95%.

### Relationships Among Outcome Measures

**Fig. 4.**
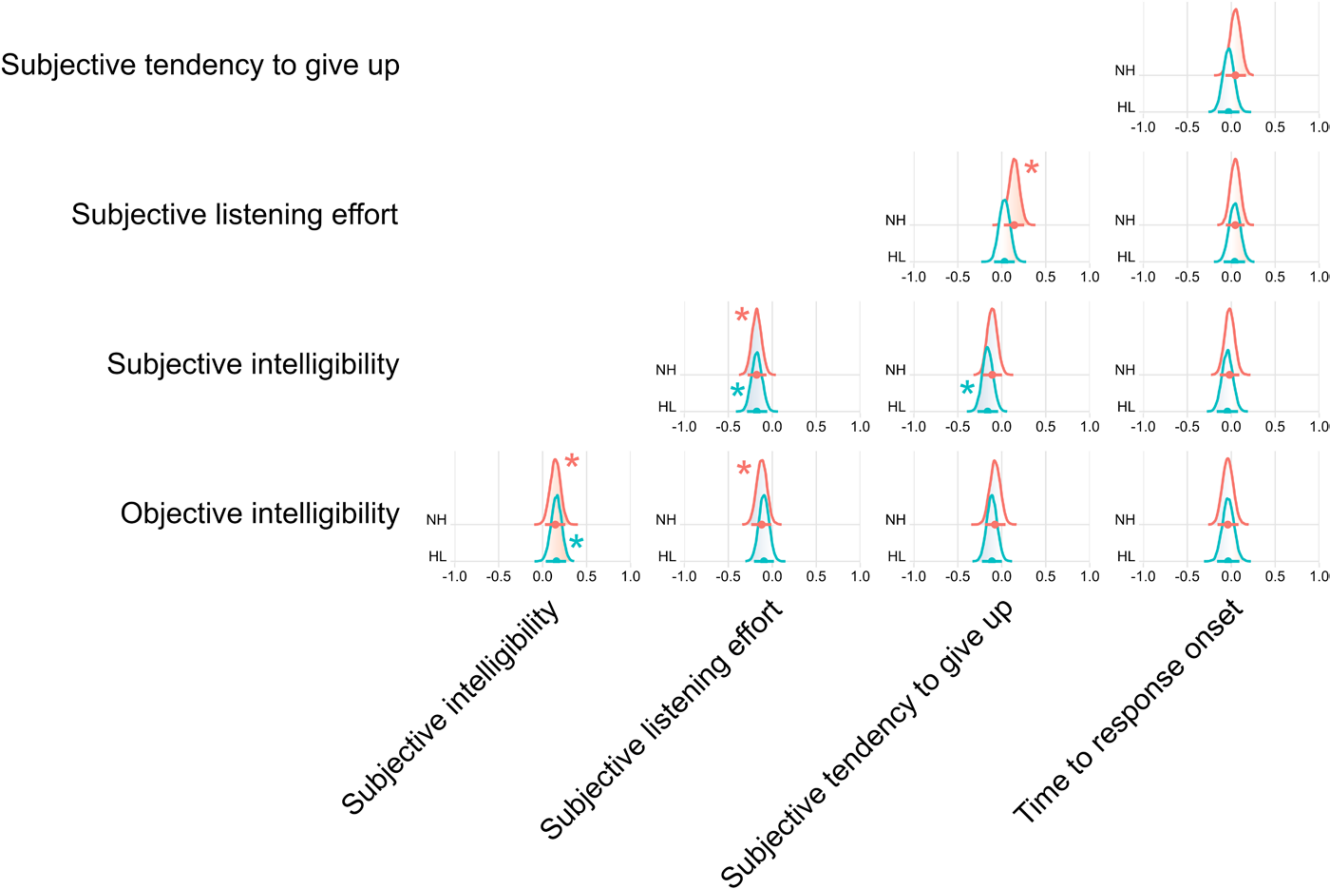
Correlations between outcome measures, as estimated from the correlated random effects included in the multivariate model (mean across SNRs). For each pair of outcome measures, the posterior distribution (plus median and 95% highest density interval) for the corresponding correlation coefficient ([−1, +1]) is plotted for the NH and HL groups. *Cases in which the credibility of a non-zero correlation coefficient was ≥95%. HL indicates hearing loss; NH, normal hearing; SNR, signal to noise ratio.

Figure 4 shows the estimated correlations (posterior distributions) between outcome measures, based on the correlated random effects included in the multivariate model. Note that duration of verbal response has been omitted from this figure (and subsequent figures in this section), because this outcome measure showed minimal sensitivity to hearing status or SNR in the primary analysis. Since random-effect hyperparameters were allowed to vary by group, the inter-measure correlations were also estimated separately within the NH and HL groups. The correlations shown in Figure 4 correspond to the mean across SNRs.

The most robust correlations were observed between objective and subjective intelligibility (positively correlated) and between subjective intelligibility and subjective listening effort (negatively correlated). In both cases, the 95% highest density interval did not include zero for both the NH and HL groups, indicating high credibility of the detected correlations. For other pairs of outcome measures, a credible correlation was found for one group, but not the other. Specifically, objective intelligibility and subjective listening effort were negatively correlated within the NH group, subjective listening effort and subjective tendency to give up were positively correlated with the NH group, and subjective intelligibility and subjective tendency to give up were negatively correlated within the HL group. It must be noted that relatively small (and unequal) within-group sample sizes may have influenced our ability to reliably detect correlations in some cases. Time to verbal response onset was found not to be robustly correlated with any of the other outcome measures, in either group. Where robust inter-measure correlations were detected, the absolute magnitude of those correlations was small (median estimates of |*r*| ~ 0.15). In part, this reflects the averaging that was performed across SNRs, because at some SNRs and for some outcome measures, there was little variability between individuals due to floor/ceiling effects, restricting the scope for strong correlations to emerge. Indeed, if analysis was instead restricted to just the −4 dB SNR condition, at which most measures exhibited substantial between-participant variability, the magnitude of the observed correlations was up to twice as large (median estimates of |*r*| ~ 0.3), although the overall pattern of the correlation matrix was unchanged.

**Fig. 5.**
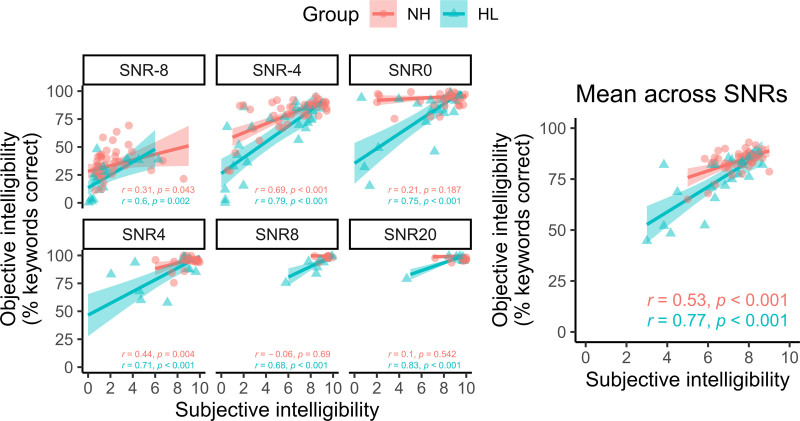
Correlations between subjective and objective intelligibility (raw scores as measured), for each SNR separately (left panels) and averaged across SNRs (right panel). HL indicates hearing loss; NH, normal hearing; SNR, signal to noise ratio.

The relationship between objective and subjective intelligibility is examined in greater detail in Figure 5. These results show that the correlation between objective and subjective intelligibility (as assessed on the raw data) was consistently weaker in the NH group than in the HL group, dramatically so at positive SNRs. Overall, the slopes of the regression lines relating objective to subjective intelligibility were shallower than the unity diagonal (after accounting for the fact that objective intelligibility was measured on a [0–100] percentage-correct scale whereas subjective intelligibility was measured on a [0–10] scale) such that participants tended to rate their perceived accuracy lower than their objective percentage-correct scores would have implied. This was especially the case for participants with NH.

**Fig. 6.**
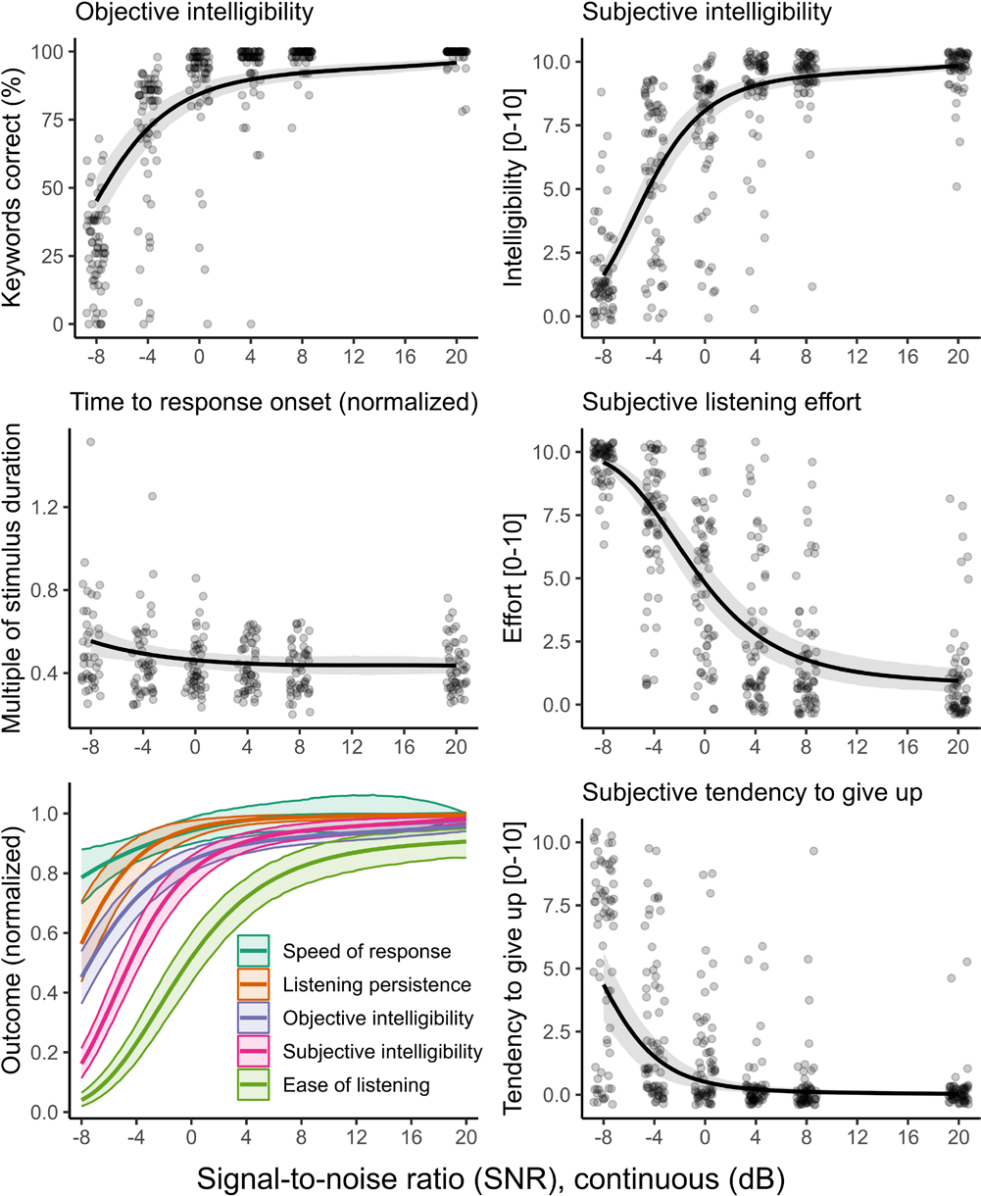
Overall dependency of each outcome measure on SNR. Black lines and shaded areas indicate the median and 95% credible intervals for the expected value of each outcome measure as a function of SNR. Raw data for individual participants are shown as small circles (shaded to indicate density of overlapping data points). For the summary plot (lower-left panel), all measures were expressed as proportions on a common [0 to 1] scale. “Ease of listening” and “Listening persistence” were defined for convenience as the inversions of subjective listening effort and subjective tendency to give up, respectively. “Speed of response” was defined as the ratio between time to response onset at a fixed reference SNR of +20 dB and time to response onset at each individual SNR. SNR indicates signal to noise ratio.

Figure 6 shows the results of the secondary analysis in which SNR was treated as a continuous variable and data (pooled across groups) were modeled using third-order polynomials. Individual panels show the results for each outcome measure separately, highlighting that, while the fitted polynomials capture group-average patterns well, interindividual variability was pronounced, especially at intermediate SNRs (e.g., −4 to +4 dB).

The bottom-left panel in Figure 6 shows the overall results for all outcome measures combined. From these results, the following pattern emerges:

- Ease of listening (the inverse of subjective listening effort) is affected earliest when moving from favorable to unfavorable SNRs (+ve to –ve). Specifically, compared with +20 dB SNR, ease of listening had already declined by around 32% by 0 dB SNR.
- The second earliest measure to respond to worsening SNR was subjective intelligibility, which lay between the curves for subjective listening effort (/ease of listening) and objective intelligibility. This corroborates the finding from Figure 5 that participants’ subjective impression of their accuracy tended to underestimate their objective, measured performance.
- For the average listener, “listening persistence” (defined as the inversion of subjective tendency to give up) declines from ceiling level only at adverse (negative) SNRs (−4 to −8 dB).
- “Speed of response” (the inversion of time to verbal response onset, exact definition in the caption to Fig. 6) was the last measure to respond to worsening SNR, showing an approximately 20% decline between the most favorable (+20 dB) and most adverse (−8 dB) SNRs tested.

## DISCUSSION

We conducted an online study involving participants with and without self-reported HL. Participants with HL (N = 25) on average reported experiencing almost twice as much listening effort in daily life as participants with NH (N = 42), although there was considerable overlap in EAS scores between groups. In an online speech-in-noise test, participants heard sentences in noise across a broad range of SNRs and then repeated back what they heard, with responses recorded via webcam. We evaluated performance using a range of objective and subjective outcome measures designed to tap into accuracy and listening effort. The outcome measures varied in their sensitivity to hearing status, SNR, and age, as well as the degree to which they correlated with one another.

### HL and Age Led to Increased Listening Difficulties

The results of the present study corroborate that both HL and age contribute to increased difficulty in understanding speech in noise (Moore et al. 2014; Von Gablenz & Holube 2017; Rahne et al. 2023). In the present study, self-reported HL was associated with poorer intelligibility (objective and subjective), greater subjective listening effort, and longer VRT compared with NH listeners. For most outcome measures, the between-group difference was most pronounced at adverse (negative) SNRs. The exception was subjective listening effort, which was consistently greater in the group with HL than the group with NH at all but the most adverse SNR (where a ceiling effect became predominant). Perhaps surprisingly, we did not observe any clear effect of hearing status on self-reported tendency to give up listening under challenging conditions, which may have been due, at least in part, to a very high level of interindividual variability in how this question was answered.

Independently of HL, older age was associated with poorer objective speech intelligibility and longer VRT. In general, subjective ratings were less influenced by age than were the objective measures, once HL was accounted for. Relatively weak effects of age on subjective ratings in the present study may relate to the finding that older listeners tend to overestimate their hearing abilities and underestimate their difficulties relative to younger listeners (Von Gablenz & Holube 2017).

### Implications for Outcome-Measure Selection and Choice of Test Conditions in Speech-in-Noise Studies

The results of the present study suggest that not all outcome measures have an equal ability to capture the effects of hearing status, age, and SNR on speech-in-noise listening experiences. In most auditory studies, the intention is to test for differences between groups of individuals, or between different conditions or devices tested on the same individuals. In such a context, the effects of age will typically be an unwanted confound, while the choice of appropriate test conditions (specifically, SNRs) becomes primarily an experimental design consideration.

Our findings indicate that speech intelligibility (objective or subjective) is likely to be a sensitive outcome measure for detecting between-group or between-condition differences, but only if testing is conducted close to the limits of performance (generally speaking, at adverse, negative SNRs). However, in the real world, listeners are exposed to such adverse acoustic conditions relatively rarely, with a majority of listening experiences occurring at moderately positive SNRs (Smeds et al. 2015; Wu et al. 2018; Weisser & Buchholz 2019). Moreover, modern digital hearing aids can be expected to behave quite differently depending on the input SNR, meaning that testing with hearing devices at strongly negative SNRs may be doubly unrealistic (Naylor 2016).

To test under more ecologically relevant SNRs (Smeds et al. 2020), a listening effort outcome measure may provide more insight. In the present study, we found that subjective listening effort was highly sensitive to self-reported HL across a broad range of SNRs, including those in which intelligibility scores were largely indistinguishable between listeners with and without HL. Compatible findings have been observed elsewhere using behavioral and physiological measures of listening effort. For example, studies using variations of the sentence-final word identification and recall test have shown that digital noise reduction can reduce listening effort, even when intelligibility remains close to ceiling level (Ng et al. 2013; Lunner et al. 2016). Similarly, Winn et al. (2015) showed that more challenging listening conditions resulted in greater pupil dilation (interpreted as more listening effort), even on trials for which intelligibility was at 100%.

Our findings suggest that, when asked to reflect on how many of a set of sentences a participant understood, there was a tendency to report a lower value than was indicated by a concurrent objective assessment of speech intelligibility. This was especially so for people with self-reported NH. An important caveat to this finding is that we asked participants how many sentences they thought they understood, whereas when assessing intelligibility objectively we scored the number of keywords that were correctly repeated. However, a supplemental analysis using sentence-level objective scoring did not substantively change this finding, and so we believe it may reflect a general pattern. This could conceivably reflect that when listeners are asked to self-report intelligibility, they are instinctively influenced by the confidence that they felt in their responses (even if those responses should turn out to be fully, or mostly, correct). An alternative interpretation is that listeners struggle to report intelligibility independently of other dimensions of the listening experience, such as perceived task difficulty or listening effort (cf. Morimoto et al. 2004). It may also be the case that listeners tend to give undue weight to a small number of perceived errors, amidst a wider set of correctly perceived sentences, when judging subjective intelligibility.

### Sentence-Level VRT as a Measure of Listening Effort

In the present study, we also examined VRT as a putative behavioral measure of listening effort. We did not find any evidence that verbal response duration (the amount of time spent physically speaking on correctly repeated trials) varied systematically with hearing status or SNR. This suggests that the cost of mental repair of initially misheard or missed parts of the speech (Winn & Teece 2022), in terms of prolonged mental computation time, was incurred during the period of reflection/rehearsal before a participant began speaking, or, perhaps less likely, that participants were able to continue mental repair processes in the background, while vocalizing their response, without this causing any prolongation of the verbal response.

In the literature, VRT has typically been defined as the time taken for a participant to begin their verbal response (time to response onset). Using this definition, our findings were broadly consistent with the idea of longer VRT being a marker for greater listening effort, in that VRT increased as the SNR became more negative, in line with previous findings (Holube 2011; Pals et al. 2015; Meister et al. 2018; Ibelings et al. 2022; Visentin et al. 2022; Gustafson et al. 2023a). In addition, the tendency was toward longer VRT in participants with HL compared with those with NH, again consistent with the literature (Holube 2011; Meister et al. 2018). However, the changes in VRT were small in magnitude compared with changes seen in other outcome measures, and a robust between-group difference in VRT was not evident at any individual SNR. We also found that VRT was not robustly correlated with any of the other outcome measures, within either the NH or HL group.

Taken at face value, our findings may suggest that VRT is less sensitive to SNR than other outcome measures. However, it is important to note that a direct comparison between VRT and the other outcome measures included in the present study may be unfair. This is because: (a) reliable response time data were missing for around one-quarter of the dataset, reducing the effective sample size for VRT analyses; and (b) we analyzed VRT from correct trials only, making it in effect a measure of residual listening effort, after factoring out any decrement in intelligibility. The exclusion of “incorrect trials” also meant that, when moving toward more adverse SNRs, there were increasingly few trials available to inform the estimates of VRT, which may have reduced statistical power. Indeed, at −8 dB SNR, when restricting VRT analyses to correct trials only, there were on average only 3.2 out of 16 trials available to inform the estimate of VRT. As with other variables, VRT might have been more sensitive at adverse SNRs, but we had little data available for analysis.

**Fig. 7.**
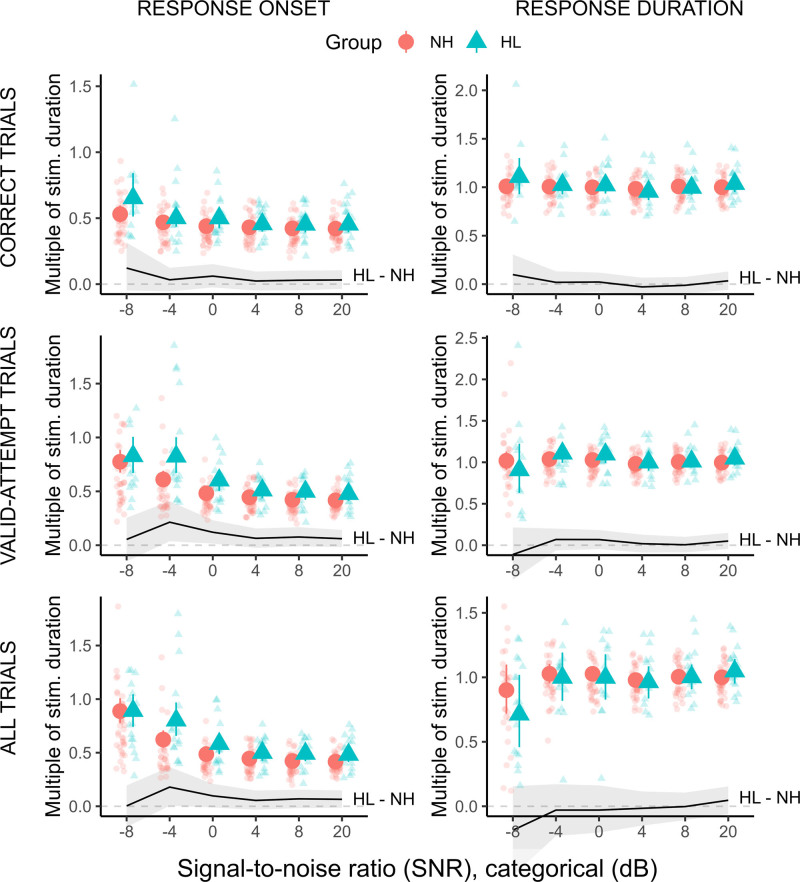
Effect of varying VRT trial-inclusion criteria: Time to verbal response onset (left column) and verbal response duration (right column) assessed across correct trials only (top row; as in the primary analysis reported elsewhere in the article), “valid-attempt” trials (middle row; attempt made to recall at least one sentence keyword), or all trials (bottom row). All other symbols and details are as in Figure 2. HL indicates hearing loss; NH, normal hearing; SNR, signal to noise ratio; VRT, verbal response time.

To explore whether different trial-inclusion criteria would have affected our VRT analyses, we conducted supplemental analyses in which VRTs were assessed using two alternative approaches: (i) inclusion of all “valid-attempt” trials, on which a participant attempted to recall at least one sentence keyword; (ii) inclusion of all trials, irrespective of the nature or accuracy of the response. The results are shown in Figure 7, with the original approach of assessing VRTs on correct trials only included for comparison. As might be expected, the general pattern was for the spread of data points to increase as the trial-inclusion criterion was relaxed and a greater diversity of verbal responses entered the analysis. For example, on some trials, a participant may simply have responded “Don’t know,” possibly quickly, or possibly after having spent some time silently rehearsing the stimulus. This spreading of VRT distributions was most evident at negative SNRs.

For verbal response duration, there continued to be no clear effect of SNR, nor between-group difference, regardless of how VRTs were calculated (except for an apparent shortening of average response duration at the most adverse SNR as increasingly more “incorrect” trials were allowed into the analysis). For time to verbal response onset, there were subtle differences depending on how VRTs were calculated. The most prominent effect was that the increase in time to response onset at negative SNRs became increasingly steep as the trial-inclusion criterion was relaxed. When VRTs were calculated across all trials, the relative increase in expected time to response onset between the most favorable SNR (+20 dB) and the most adverse SNR (−8 dB) was 85% (HL group) to 113% (NH group). Furthermore, unlike when VRT was calculated for correct trials only, more relaxed trial-inclusion criteria meant that a clear (credibility of effect ≥95%) between-group difference (HL > NH) in time to response onset could be detected at specific SNRs (most prominently at −4 dB SNR, but also at 0, 8, and 20 dB SNR). However, at −8 dB SNR, as the trial-inclusion criterion was relaxed, the between-group difference appeared to become smaller, not larger, suggesting a complex interplay between data and analysis approach. It may be that there is a trade-off between allowing more data into the analysis (increasing statistical power) versus allowing increasingly diverse verbal responses to influence the results (reducing statistical power). A sensible starting point for future studies might be to include in VRT assessments all trials on which a participant made a genuine attempt at repeating at least part of the target speech.

Taking the above findings all together, we believe that VRT may indeed be a useful outcome measure for inclusion in future speech-in-noise studies, though with the caveat that we found VRT to be in general quite a noisy measure and one that was strongly influenced by age. It may be the case that VRT has better reliability when used with single-word stimuli as in Gustafson et al. (2023b) than with sentence-length stimuli (as in the present study), due to the greater diversity of verbal responses that can be expected with more complex stimuli. To the best of our knowledge, this remains to be examined empirically.

### Limitations

Several limitations of the present study should be acknowledged. First and foremost, the online nature of the study meant that we were unable to control for differences in device, operating system, browser, sound reproduction hardware, volume settings, and listening environment across participants, beyond some basic instructions and sanity checks issued at the start of the online experiment. Nevertheless, it is improbable that these variables changed substantially during the course of an individual’s participation, offering within-subject control to the assessment of the effect of varying SNR. A further limitation relating to the online context was that we had to rely on self-report to stratify participants into the NH and HL groups, giving rise to the possibility that some participants in the NH group may in fact have had undiagnosed hearing impairments.

A second limitation of this study was that the overall sample size was relatively modest (N = 67), limited by the available timeframe and funding for data collection, resulting in unequally sized and non–age-matched NH and HL groups. We were able to overcome these limitations to a certain extent through the use of Bayesian statistical methods, which are less sensitive to unequal group sizes, and by including age as a covariate within our primary statistical models.

Finally, we note that the 0.5 seconds of background noise that preceded the sentence onset on each trial may not have been sufficiently long for any noise reduction algorithms to have fully engaged and stabilized in those participants (N = 13) who used a hearing aid (/aids) or cochlear implant. In this regard, our results might conceivably underestimate the performance that such individuals would have achieved with their hearing devices under steady state conditions. We would not expect this to have affected the interrelations between outcome measures—a main focus of the study—although it could have overestimated the differences seen between the NH and HL groups.

## CONCLUSION

Despite the challenges and uncertainties of delivering auditory research in an online setting, the present findings offer useful insight into how different subjective and objective measures of listening accuracy and effort respond to variation in hearing status, age, and SNR. Although speech intelligibility remains a measure of primary importance, its usefulness is limited to more adverse listening conditions, which may not be representative of everyday listening experiences. Under more ecologically relevant listening conditions (generally speaking, at moderate, positive SNRs), listening effort becomes a crucial factor to consider to adequately describe the listening experience. VRT may provide a useful objective marker of listening effort, but caution is required to deal with measurement variability, differences in definition, and the potentially confounding effect of age.

## ACKNOWLEDGMENTS

The authors thank Sandra Smith for help with participant recruitment, and Diana Zaitseva and Ben Rogers for data coding.

## Supplementary Material



## Footnotes

* The participant’s response was taken as beginning with the onset of the first word uttered (following the auditory cue) and ending with the offset of the last word uttered; any other sounds (coughs, sighs, humming, etc.) occurring before or after the verbal response were ignored and therefore excluded from the marked response period.

† Keyword accuracy was scored using a relatively forgiving protocol in which errors of tense and/or plurality were ignored, so long as the basic meaning of the sentence was preserved.
