# Supplementary material for: Relationships Between Subjective and Objective Measures of Listening Accuracy and Effort in an Online Speech-in-Noise Study
Source: Ear Hear. 2025 Mar 21;46(5):1197–209. doi: 10.1097/AUD.0000000000001662 (PMC12352569; doi:10.1097/AUD.0000000000001662)
Supplement: Supplementary file 1 [file aud-46-1197-s001.pdf]

Supplemental Digital Content 1 – Demographics and Hearing Health Questionnaire Items

Enter your age in years into the box below

Please select your sex at birth

- ○ Male
- ○ Female

How is your hearing in your <u>left</u> ear?
(If you are unsure, please select the option that you feel best describes your hearing in this ear)

	Good – no loss	Mild loss	Moderate loss	Severe loss	Profound loss
Left ear	○	○	○	○	○

Do you use a hearing device in your <u>left</u> ear?

	None	Hearing aid	Cochlear implant	Other
Left ear	○	○	○	○

If you selected other please write the name of the device you use in your left ear in the box below

How is your hearing in your <u>right</u> ear?
(If you are unsure, please select the option that you feel best describes your hearing in this ear)

	Good – no loss	Mild loss	Moderate loss	Severe loss	Profound loss
Right ear	○	○	○	○	○

Do you use a hearing device in your <u>right</u> ear?

	None	Hearing aid	Cochlear implant	Other
Right ear	○	○	○	○

If you selected other please write the name of the device you use in your right ear in the box below
