# Supplementary material for: Relationships Between Subjective and Objective Measures of Listening Accuracy and Effort in an Online Speech-in-Noise Study
Source: Ear Hear. 2025 Mar 21;46(5):1197–209. doi: 10.1097/AUD.0000000000001662 (PMC12352569; doi:10.1097/AUD.0000000000001662)
Supplement: Supplementary file 2 [file aud-46-1197-s002.pdf]

Supplemental Digital Content 2

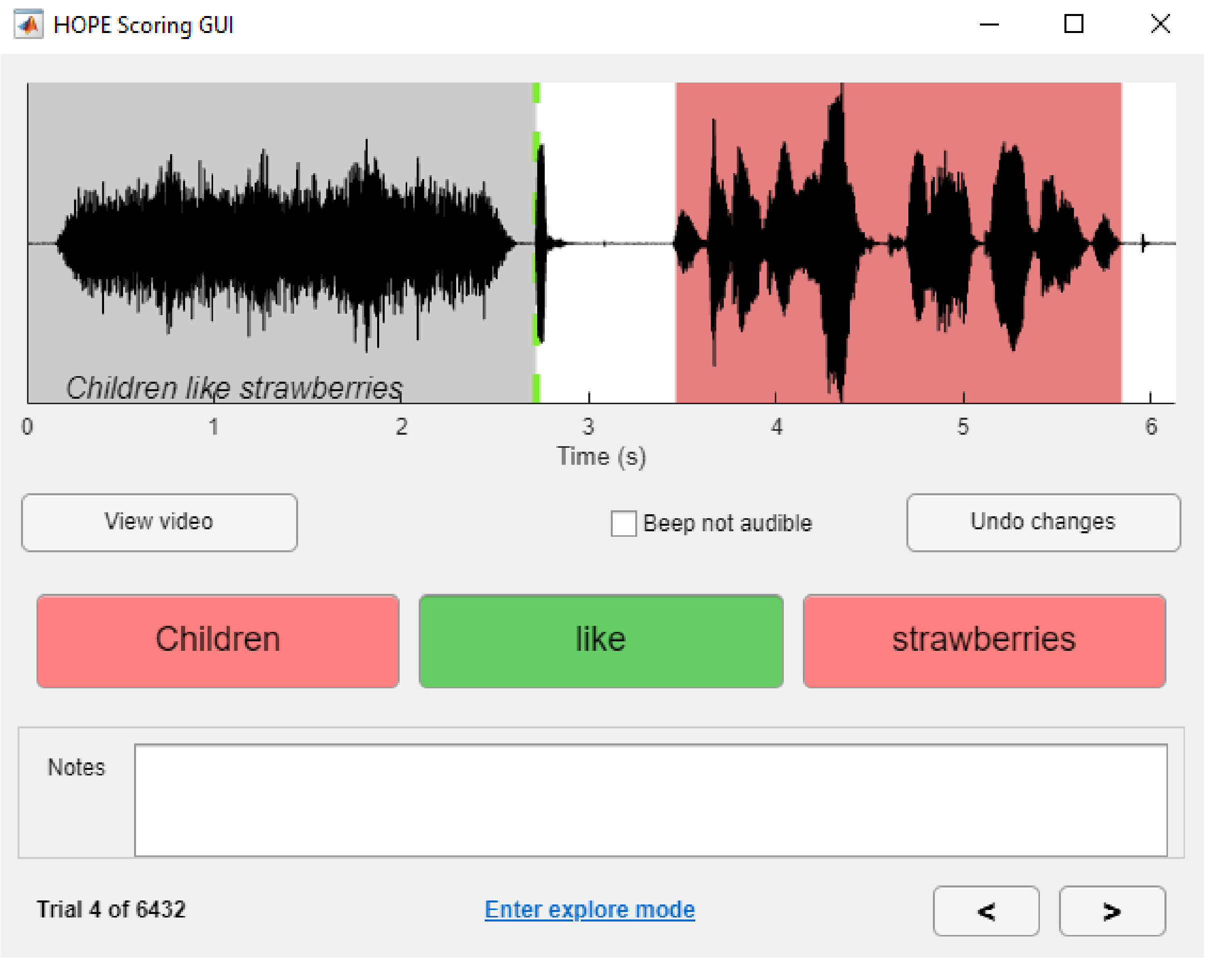

Figure. Screenshot of the custom MATLAB scoring app developed to facilitate data analysis. The top panel shows the waveform of the webcam recording for the current trial. The pre-recorded stimulus appears to the left, followed by the auditory cue (marked by the vertical dashed green line), and then the participant's verbal response (contained within the red shaded area). The position of the audio markers could be adjusted using the mouse scroll wheel. In the lower half of the GUI, scorers could use toggle buttons to mark keywords as correct or incorrect and add any notes. The video recording for the current trial could be opened in a separate window as needed to assist keyword scoring.
