# Supplementary material for: Relationships Between Subjective and Objective Measures of Listening Accuracy and Effort in an Online Speech-in-Noise Study
Source: Ear Hear. 2025 Mar 21;46(5):1197–209. doi: 10.1097/AUD.0000000000001662 (PMC12352569; doi:10.1097/AUD.0000000000001662)
Supplement: Supplementary file 3 [file aud-46-1197-s003.pdf]

# Supplemental Digital Content 3 – BRMS model formulae

```
bf_KeywordsCorrect <- bf(KeywordsCorrect | trials(KeywordsTotal) + subset(IncludeInKeywordsCorrectModel) ~
                        Group*SNR + Age + (0 + SNR|gr(ParticipantID, id = "corrID", by = Group)),
                        family = beta_binomial())

bf_Q1_LE <- bf(Q1_LE_scaled | subset(IncludeInVASModels) ~
                  0 + Intercept + Group:SNR + Age + (0 + SNR|gr(ParticipantID, id = "corrID", by = Group)),
                  family = ord_beta_reg)

bf_Q2_intel <- bf(Q2_intel_scaled | subset(IncludeInVASModels) ~
                  0 + Intercept + Group:SNR + Age + (0 + SNR|gr(ParticipantID, id = "corrID", by = Group)),
                  family = ord_beta_reg)

bf_Q3_giveup <- bf(Q3_giveup_scaled | subset(IncludeInVASModels) ~
                  0 + Intercept + Group:SNR + Age + (0 + SNR|gr(ParticipantID, id = "corrID", by = Group)),
                  family = ord_beta_reg)

bf_ResponseOnset <- bf(ResponseOnsetAsMultipleOSD | subset(IncludeInVRTModels) ~
                        Group*SNR + Age + (0 + SNR|gr(ParticipantID, id = "corrID", by = Group)),
                        sigma ~ Group*SNR,
                        family = lognormal())

bf_ResponseDuration <- bf(ResponseDurationAsMultipleOSD | subset(IncludeInVRTModels) ~
                        Group*SNR + Age + (0 + SNR|gr(ParticipantID, id = "corrID", by = Group)),
                        sigma ~ Group*SNR,
                        family = lognormal())

fit <- brm(bf_KeywordsCorrect + bf_Q1_LE + bf_Q2_intel + bf_Q3_giveup + bf_ResponseOnset + bf_ResponseDuration +
            set_rescor(FALSE),
      data = df_mv,
      …
      …
      backend="cmdstanr")
```
